# On the Conservation of Force in Disease

**Published:** 1868-09

**Authors:** J. E. Hendricks

**Affiliations:** Des Moines, Iowa


ARTICLE XXXVI.

### ON THE CONSERVATION OF FORCE IN DISEASE.

By J. E. HENDRICKS, M.D., Des Moines, Iowa.

The investigations of philosophers, during the last 20 years, have placed the doctrine of the *Conservation of Forces* on so firm a basis that its universality may be considered as *demonstrated.* All physiological and pathological conditions may, therefore, safely be attributed to some prior manifestation of *Force.** A definite amount of force, which is ultimately traceable to ethereal vibrations,† results in the construction of animal tissue. This tissue, besides being an *instrument* through which other forces may be manifested, is itself a force, *exactly* equivalent to the force by which it was produced. The admission of the conservation of forces makes this equivalence *absolutely* necessary.

If, therefore, animal tissue disappears during disease, we *know* that either the tissue has been imperfectly disorganized, and yet represents a portion of the force which disappeared on its organization; or its total equivalent has been manifested in some form, if not in its usual form of "psychical and motor" force, then in the form of *heat*, or some other occult form that has escaped our observation.

It is illogical, therefore, to conclude, when animal tissue is

* Force is comprehensible only as a result or consequence of, and coëxistent with, motion. It is either *active* or *potential.* Animal tissue, when not in a state of retrograde metamorphosis, is a representation of *potential* force. The *motion* here is not apparent, but the persistence of force *necessitates* the continuance, even here, of (molecular) motion.

† That heat is a form of force having a definite mechanical equivalent, has been abundantly demonstrated. And it is now pretty generally believed by the more advanced philosophers, that *heat, light, electricity, magnetism, chemical attraction*, and perhaps *gravitation*, are all "convertible material affections." All these affections, or forms of force, except, perhaps, the latter, are ultimately traceable to *ethereal undulatory motion.* That the latter, or the *attraction of gravitation*, is referable to the same cause, ethereal vibratory motion, although not so obvious, is, nevertheless, highly probable.

disorganized and eliminated in disease without producing the usual amount of mental and motive force, or any observable form of force except *heat*, that the *tissue* was in any respect defective or incapable of conversion, by proper organisms, into a full equivalent of its usual forms of force. To conclude thus, is, in fact, to deny the universality of the conservation of forces.

As the various forms of force manifested by living animal organizations are ultimately traceable, mainly, to that form of force we call heat, the modified forms being the result of organisms, it is, therefore, highly probable, *a priori*, that if those organisms are by any means deranged, the manifestation of force through them would be changed; and, though for a given amount of disintegrated tissue a given amount of force must result, yet in the above supposed condition of the organisms that force may *all* be manifested as heat.

In some diseases, where certain tissues are rapidly dissolved and carried out of the system (as in cholera, for instance), it is probable that but a *partial* retrograde metamorphosis has been effected, and that the tissue, in its dissolved state, contains a considerable portion of the force expended in its organization. This residue of force, in that case, would only be manifested as heat upon *putrefaction* of the fluids after their elimination. It is highly probable, therefore, that in every case of disintegrated tissue, if we could follow it through its successive changes, until complete disorganization is effected, and could measure all the manifestations of force resulting therefrom, we would discover that their *sum* is exactly an equivalent of the force that had been expended in its organization. Any other view of the case is, virtually, a denial of the doctrine of the conservation of forces.

